# A biochronological date of 3.6 million years for “Little Foot” (StW 573, *Australopithecus prometheus* from Sterkfontein, South Africa)

**DOI:** 10.1002/evan.22049

**Published:** 2024-11-01

**Authors:** Francis Thackeray

**Affiliations:** ^1^ Evolutionary Studies Institute University of the Witwatersrand Johannesburg South Africa

**Keywords:** *africanus*, *Australopithecus*, Pleistocene, pliocene, *prometheus*, South Africa, Sterkfontein

## Abstract

A debate has developed with regard to geological ages of hominin fossils attributed to *Australopithecus africanus* and *Australopithecus prometheus* in South African Plio‐Pleistocene cave deposits. For the Sterkfontein caves (Members 2 and 4), cosmogenic nuclide isochron (^10^Be/^26^Al) dating has yielded age estimates ranging from 3.4 to 3.7 million years ago (Ma). However, biochronological approaches using nonhominin primates suggest an alternative age range between 2 and 2.6 Ma. Based on a new method of hominin biochronology, Thackeray and Dykes have recognized that Sterkfontein Member 4 has a mean age of 2.76 Ma associated with a wide range (circa 2.0–3.5 Ma). In this study, the Sterkfontein skull and skeleton (StW 573), nicknamed “Little Foot” from Member 2 and attributed to *A. prometheus*, is reassessed. A regression model applied to estimate its age provides a hypothesized date of 3.6 Ma, which compares favorably with the existing cosmogenic dates.

## 1 INTRODUCTION

*Australopithecus africanus* and *Australopithecus prometheus* are South African Plio‐Pleistocene hominin species considered key to understanding early human evolution. A significant debate has emerged regarding the geological ages of these fossils, particularly concerning their attribution to different stratigraphic layers within South African cave deposits. In the case of the Sterkfontein caves (Members 2 and 4), one group of researchers^1^, ^2^ places them between 3.4 and 3.7 million years ago (Ma) based on cosmogenic (^10^Be/^26^Al) dating. However, biochronological methods using a diversity of nonhominin primates place these fossils between 2 and 2.6 Ma.^3^ Counter‐criticisms from both sides have been presented.^4^, ^5^ For example, Granger et al.^4^ noted instances of misidentification between hominin and nonhominin fauna such as *Equus*, complicating the interpretation of the fossil record. More recently, Thackeray and Dykes,^6^ using a biochronological approach, suggested that Sterkfontein Member 4 is dated to 2.76 Ma, associated with a wide range from circa 2.0–3.5 Ma.^6^, ^7^, ^8^ In this study we reassess the Sterkfontein skull and skeleton (StW 573) nicknamed “Little Foot” from Member 2, attributed to *A. prometheus*.^9^, ^10^, ^11^, ^12^ The goal is to try to answer the question as to whether its age is 3.67 ± 0.16 Ma^1^ or if it falls below 2.6 Ma. A hypothesis is presented regarding its date.

## 2 BACKGROUND TO DISCOVERIES

**Figure 1:**
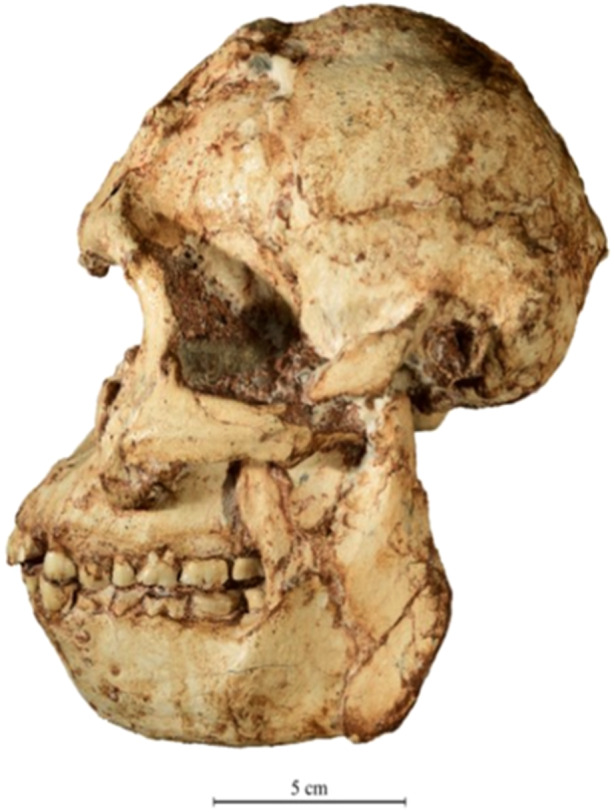
Left lateral view of the adult australopithecine skull of “Little Foot” (*Australopithecus prometheus*, StW 573), as published by Clarke and Kuman.^12^ *Source*: Photograph by Matt Lotter, courtesy of R. J. Clarke.

The first discovery of a Plio‐Pleistocene specimen of *Australopithecus africanus* occurred in 1924 at Buxton‐Limeworks near Taung, South Africa. The juvenile cranium, known as the “Taung Child,” was described by Raymond Dart,^13^ a professor of anatomy at the University of the Witwatersrand in Johannesburg. A decade later, Robert Broom^14^ discovered the first adult cranium of *A. africanus* at Sterkfontein in Gauteng, approximately 70 km southwest of Pretoria.^15^ This was followed by post‐war discoveries with John Robinson. Both paleontologists were based at the Transvaal Museum (now the Ditsong National Museum of Natural History in Pretoria). A notable skull of *A. africanus* (Sts 5) found by them was the celebrated “Mrs Ples.”^16^ In 1966, Phillip Tobias (Wits) resumed excavations at Sterkfontein with Alun Hughes, and from 1991 with Ron Clarke. This led to the dramatic discovery of “Little Foot” (Figure 1), an exceptionally complete hominin skeleton^17^, ^18^ whose precise age remains controversial.

Fossils described initially by Dart^19^ as *A. prometheus* had been found at Makapansgat in the Limpopo Province. Among paleoanthropologists, there is no consensus as to whether *A. prometheus* is an australopithecine species distinct from *A. africanus*. In this study, the former nomen is preferred for StW 573, in accordance with Clarke,^9^, ^10^ Clarke et al.,^11^ as well as Clarke and Kuman.^12^

## 3 BACKGROUND TO CHRONOLOGY

Dating South African australopithecine fossils is challenging because early discoveries, often made during calcite mining, were disturbed by dynamite such that context was lost. In East African contexts, radiometric dating (e.g., potassium‐argon) is used, but its absence in South Africa has necessitated alternative methods like biochronology. Efforts have been made to obtain biochronological estimates of ages for South African Plio‐Pleistocene fauna^20^ including suids such as warthogs,^21^ bovids including antelope such as wildebeest, hartebeest and kudu^22^ in addition to cercopithecid monkeys.^3^, ^5^, ^23^ Attempts have been made to use paleomagnetism and uranium‐lead ratios^24^, ^25^, ^26^, ^27^, ^28^, ^29^ as well as cosmogenic nuclides.^1^, ^2^, ^4^ In response to a stratigraphic scenario for Sterkfontein Members 2, 3, and 4,^30^ a comprehensive analysis^31^ was published to address the chronological complexities in these deposits.

Table 1 summarizes the range of dates proposed for Sterkfontein Members 2 and 4, highlighting the various biochronological and radiometric methods used. It should be noted that dates obtained by Frost et al.^3^, ^5^ for Member 4 (2.0–2.6) correspond closely to those reported by Herries et al.^27^ but the Member 2 flowstones analyzed by the latter were intrusive.^4^, ^31^

**Table 1:** Dates for Sterkfontein for Member 4 (M4) and Member 2 (M2).

Range (Ma)	Mean	Reference of range
3.67 ± 0.16	3.67	Granger et al.,^1^ cosmogenic nuclides (M2)
—	3.6	Thackeray (this study), biochronology (M2), StW 573
3.4–3.7	3.55	Granger et al.,^2^, ^4^ cosmogenic nuclides (M4)
3.22–3.58	3.40	Partridge et al.,^24^ paleomagnetism (M2)
2.07–3.85	2.96	van Holstein and Foley,^32^ Time‐Based Variability (TBV) model
1.70–4.33	3.01	van Holstein and Foley,^32^ Within Lifetime Variability (WLV) model
2.5–3.0	2.75	Cooke,^21^ suids (M4).
1.8–3.5	2.65	Thackeray,^7^ biochronology (M4).
2.3–2.8	2.55	Vrba,^22^ bovids (M4)
—	2.4	Delson,^23^ cercopithecids (M4)
2.05–2.58	2.31	Herries et al.,^27^ paleomagnetism and U/Pb (M2, M4)
2.0–2.6	2.30	Frost et al.,^3^, ^5^ cercopithecids (M2, M4).
<3.0	<3.0	Berger et al.,^20^ fauna (M2)
<2.8 Ma	<2.8	Kramers and Dirks,^30^ scenario (M2)
>2.2	>2.2	Bruxelles et al.,^31^ stratigraphy, geochemistry (M2)
2.17–2.24	2.20	Walker et al.,^25^ U/Pb (M2)
1.5–2.5	2.0	Berger et al.,^20^ fauna (M4)

## 4 METHOD

This study applies a biochronological method used in previous studies^6^, ^7^, ^8^ to hominins by exploring the ratio of mesiodistal (MD) and buccolingual (BL) diameters of mandibular first molars (M₁). By adopting this established approach, the study aims to estimate the geological age of StW 573. Using least squares linear regression the relationship between MD/BL ratios and geological ages of well‐dated East African specimens has been established. Specimens attributed to *Australopithecus* and Early *Homo*, including *Australopithecus afarensis*, *Homo habilis*, *Homo rudolfensis*, and *Homo erectus*, were measured for purposes of quantifying MD/BL ratios, with *Paranthropus* excluded due to its distinct evolutionary lineage. The regression equation^6^, ^7^ for East African *Australopithecus* and Early *Homo* specimens is provided below, based on data in Table 2:

(1) $$\text{Geological}\;\text{age}\;\left(\text{Ma}\right)=-10.407\left(\text{MD}/\text{BL}\right)+13.990\;\left(r^{2}=0.85\right),$$

where −10.407 is the coefficient *m* in general equations of the form *y = mx* + *c*, and where 13.990 is the intercept (*c*).

**Table 2:** Radiometric dates (Ma) for lower first molars of East African specimens, compiled from published literature.^33^

Catalog ID	Site	Taxon	MD/BL	Age (Ma)
LH 4 R	Laetoli	*Australopithecus afarensis*	1.004	3.77
LH 4 L	Laetoli	*A. afarensis*	1.044	3.77
AL 145 35 L	Hadar	*A. afarensis*	0.998	3.35
AL 266‐1 R	Hadar	*A. afarensis*	1.008	3.20
AL 333 W6D L	Hadar	*A. afarensis*	1.012	3.20
AL 228 23 R	Hadar	*A. afarensis*	1.053	3.18
AL 266‐1 L	Hadar	*A. afarensis*	1.059	3.20
ER 1802 R	East Turkana	*Homo rudolfensis*	1.169	1.87
ER 1802 L	East Turkana	*H. rudolfensis*	1.194	1.89
ER 992 R	East Turkana	*Homo erectus*	1.165	1.49
ER 992 L	East Turkana	*H. erectus*	1.194	1.49
ER 820 R	East Turkana	*H. erectus*	1.184	1.60
ER 820 L	East Turkana	*H. erectus*	1.209	1.60
OH 22 R	Olduvai	*H. erectus*	1.177	0.88
OH 16 R	Olduvai	*Homo habilis*	1.156	1.74
OH 7 R	Olduvai	*H. habilis*	1.199	1.84
OH 7 L	Olduvai	*H. habilis*	1.195	1.84

*Note*: Mesiodistal (MD) and buccolingual (BL) ratios were calculated by Dykes^33^ from original specimens (Kenya and Tanzania) or high‐quality casts (Ethiopia).

Knowing MD/BL ratios for South African hominin molars of *Australopithecus* and Early *Homo* (including those cataloged by Moggi‐Cecchi et al.^34^), it is assumed that the same equation can be applied to estimate ages for hominins of the same genera in the African subcontinent.^6^, ^7^ The method is analogous to that which has been applied to nonhominin fauna from East and South Africa. Notably, Frost et al.^3^, ^5^ used tooth area of molars of *Theropithecus*, whereas in studies by Thackeray and Dykes^6^ the ratio of length (MD) and breadth (BL) of M_1_ is applied to hominins.

## 5 MATERIALS

**Figure 2:**
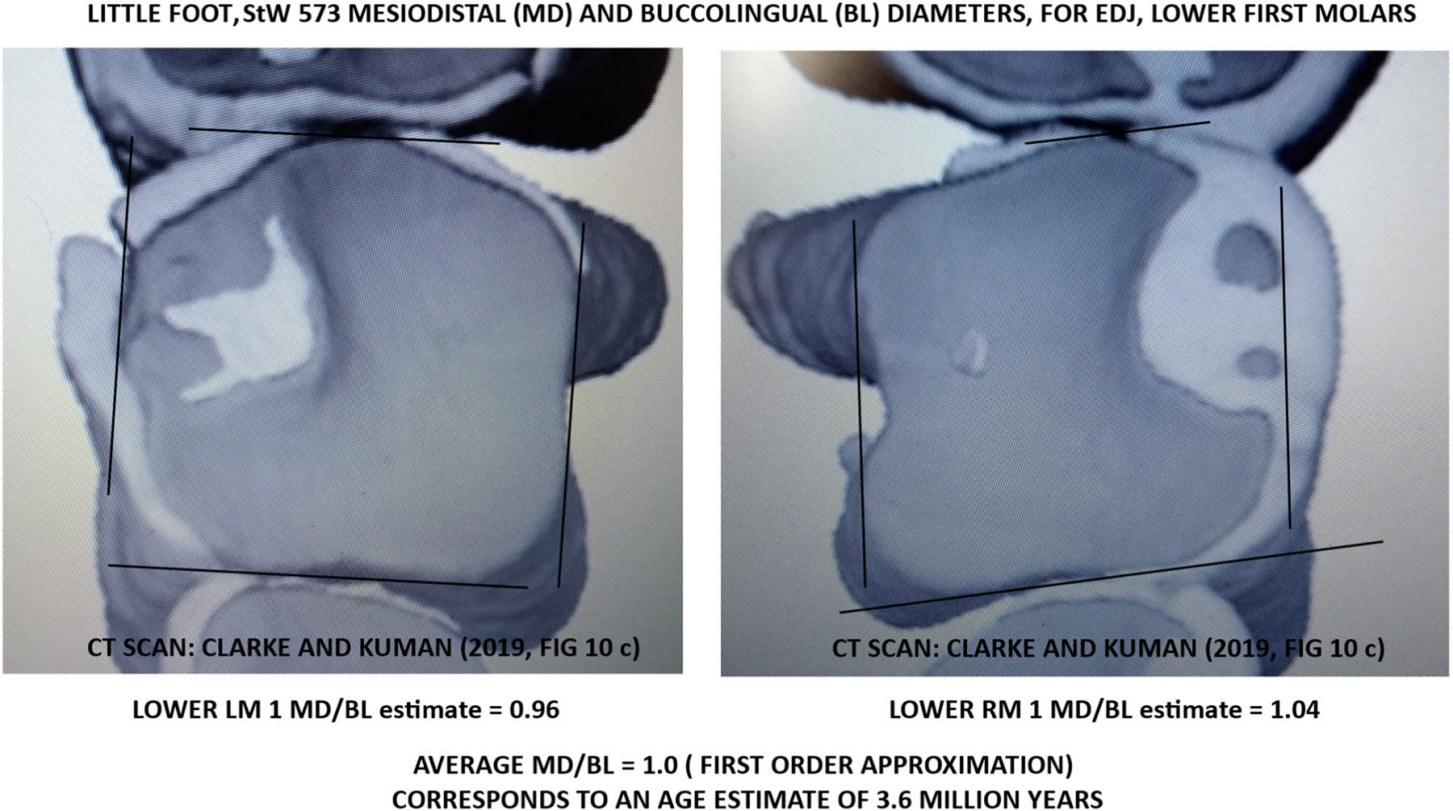
CT scans of left and right lower first molars of StW 573 (from Clarke and Kuman,^12^ their fig. 10c), indicating reference lines for MD and BL dimensions estimated from the enamel‐dentine junction (EDJ). BL, buccolingual; CT, computed tomography; MD, mesiodistal.

MD and BL measurements could not be obtained directly from the first molars of StW 573 because the maxilla and mandible are calcified together, necessitating the use of computed tomography (CT) scans (Figure 1). Micro‐CT tomographic scans of the mandibular dentition of StW 573 (published by Clarke and Kuman,^12^ their fig. 10c) were used for this study. Attention is focused on lower first molars. Due to dental wear, MD/BL ratios were calculated from measurements at the enamel‐dentine junction (Figure 2), serving as a proxy for conventional external enamel surface measurements of MD and BL.

## 6 RESULTS

Using Equation (1), a new biochronological result for StW 573 is 3.6 Ma, based on a mean MD/BL ratio of 1.0 as a first‐order approximation from EDJ measurements (Figure 2).

## CONCLUSIONS

The result of 3.6 Ma for Little Foot (StW 573), attributed to *A. prometheus* from Sterkfontein Member 2, falls within one standard deviation of a mean age of 3.67 ± 0.16 Ma based on the cosmogenic dating method of Granger et al.^1^ The result of 3.6 Ma is presented here as a hypothesized absolute age, to be tested through further research.

Dating *Australopithecus* in South Africa presents challenges, particularly due to stratigraphic disruptions from early limestone mining at Sterkfontein and other sites.^15^ Such challenges might be overcome if reliable dates can be obtained from individual hominin molar teeth, though the precision of the biochronological method remains uncertain without validation against independently dated specimens. An indication of reliability can be seen in Thackeray's^8^ dates of 2.14 and 1.93 Ma for *Australopithecus sediba* from Malapa, notably for specimens MH 1 and MH 2, respectively, where the relevant ratios are based on MD and BL measurements published by Irish et al.^35^ These results align remarkably well with independent uranium‐lead and palaeomagnetic dates pointing to an age of 1.98 Ma.^36^ The underlying message is that the hominin biochronological method developed by Thackeray and Dykes^6^ is reliable in this particular instance, with the expectation that it can be applied to other Plio‐Pleistocene specimens of *Australopithecus* in South Africa. For example, Thackeray^7^ presented an age of 2.58 Ma for the Taung Child, the holotype of *A. africanus*.^13^ Estimates for MLD 2 and MLD 40 from Makapansgat (attributed to *A. prometheus* or *A. africanus*) are 3.07 and 3.00 Ma, respectively.^6^, ^7^

The ages of 3.6 Ma for Little Foot from Member 2, and circa 2 Ma for Sts 9 from Member 4 (generally considered to represent *A. africanus* but recently attributed to early *Homo* ^37^) as published by Thackeray,^7^ illustrate the application of a biochronological approach to Sterkfontein hominins.^6^, ^7^, ^8^ They encompass almost all of the dates given in Table 1 for Sterkfontein, including those given by Granger et al.^2^, ^4^ and Frost et al.^3^, ^5^ The new biochronological estimates align with the broader debate over the geological ages of *Australopithecus*, as reflected in the differences between cosmogenic nuclide dating and nonhominin biochronological approaches. This highlights the complexities involved in dating South African hominin sites.

From the current study, it would appear that dates of circa 2.0 and 3.6 Ma may respectively be close to the upper and lower extremes for the ages of *Australopithecus* in South Africa, generally consistent with dates for *A. africanus* if not also *A. prometheus* based on models presented by van Holstein and Foley.^32^

### Future studies

Thackeray^7^ as well as Thackeray and Dykes^6^ have demonstrated an approach for dating individual hominin teeth to supplement ages determined from deposits in which the fossils are located, in addition to dates from a diversity of nonhominin taxa. In future research, going well beyond the scope of the current study, the method's accuracy and precision can be tested rigorously through cross‐validation and comparison with other dating techniques.

In the case of StW 573, it is recognized that there is extreme occlusal wear affecting enamel of the first mandibular molars, hence the need to use MD and BL measurements from the enamel‐dentine junction (Figure 2). In general, the degree to which MD/BL ratios for the EDJ correspond to those for external enamel can be assessed in future studies based on micro‐CT scans of many well‐preserved reference specimens.

Measurements from large samples of East African *Australopithecus* (including *Australopithecus anamensis*) and Early *Homo* will allow for the establishment of new equations of the kind expressed here only for lower first molars. With the use of well‐dated East African specimens including M_2_, M_3_, M^1^, M^2^, and M^3^, regression equations can be obtained by quantifying relationships between MD/BL and age for each molar tooth (not only M_1_), based on the form *y* = *mx* + *c*, where *y* is age (Ma), and *m* refers to the MD/BL ratio.

With regard to StW 573 in the case of M_2_, M_3_, M^2,^ and M^3^ it is possible to obtain MD and BL measurements from micro‐CT scans of external enamel. Using MD/BL ratios for these molars, and taking advantage of well‐dated East African samples to determine regression equations of the kind given by Equation (1), it will be possible to obtain additional age estimates for StW 573 without having to rely on measurements based on the EDJ as in the case of M_1_. It is in such ways that the hypothesized date of 3.6 Ma for Little Foot can be tested. Detailed statistical analyses would be undertaken to establish ranges of age estimates associated with 95% confidence limits. The hypothesized date of 3.6 Ma in the present study is based simply on a first‐order approximation (1.0) for the MD/BL ratio of the M_1_ of StW 573.

## CONFLICT OF INTEREST STATEMENT

The authors declare no conflict of interest.

## Data Availability

CT scan of left and right M1 of StW 573 published by Clarke and Kuman (Reference #12, their fig. 10c). Dates for individual Member 4 teeth of Australopithecus, based on MD/BL ratios for M1 specimens, have been published by Thackeray (Reference # 7).
